# Practice of balanced scorecard implementation and its contributing factors among public primary hospital professionals in Central Gondar zone, Northwest Ethiopia

**DOI:** 10.3389/fpubh.2024.1424133

**Published:** 2025-01-20

**Authors:** Seid Yeshaw, Geta Asrade, Asebe Hagos, Muluken Genetu Chanie, Nigusu Worku

**Affiliations:** ^1^Central Gondar Zone Health Department Head, Gondar, Gondar, Ethiopia; ^2^Department of Health Systems and Policy, Institute of Public Health, College of Medicine and Health Sciences, University of Gondar, Gondar, Ethiopia; ^3^Department of Health Systems and Leadership, College of Medicine and Health Sciences, Wollo University, Dessie, Ethiopia

**Keywords:** BSC implementation, BSC implementation practice, public primary hospital, Central Gondar, Ethiopia

## Abstract

**Background:**

Globally, a Balanced Scorecard (BSC) is used to measure healthcare institutional performance. Thus, since 2010, Ethiopia has adopted and implemented BSC in all its civil service organizations. Ineffective implementation of this tool has a significant impact on the healthcare performance. The health sector’s healthcare provision, customer satisfaction, customer retention, organizational profit, changes and improvements in the healthcare delivery practice are affected by the implementation of BSC tools. However, no evidence or study indicates the implementation of BSC practice in the Ethiopian context. Thus, this study aimed to assess the magnitude of BSC implementation practice and associated factors among healthcare providers at public primary hospitals in the Central Gondar zone, Northwest Ethiopia.

**Methods:**

A mixed methods study was carried out at public primary hospitals from May 25 to June 26, 2022, in Central Gondar zone with 404 study participants. Study participants were selected using a systematic random sampling technique for the quantitative part, and 12 study subjects were chosen purposively for the qualitative part. Bi-variable and multivariable logistic regression analyses were done. The strength of associations was measured using the adjusted odds ratio (AOR) at 95% CI and a *p*-value of less than 0.05 in the multivariable analysis to declare significant factors. Thematic analysis was applied for the qualitative data using open code 4.03 version software.

**Results:**

The implementation practice of BSC was found at 48.5% (95% CI: 43.6, 53.4%) with a response rate of 95.28%. Teamwork (AOR: 2.68, 95% CI; 1.53, 4.69), organizational communication (AOR: 3.24, 95% CI: 1.79, 5.89), and availability of infrastructure (AOR: 2.03, 95% CI; 1.14, 3.64) were significantly associated to balanced scorecard implementation practice.

**Conclusion:**

The implementation practice of BSC was poor compared to the national standard and the findings of prior studies. Thus, concerned stakeholders need to focus on improving and working on organizational communication channels, teamwork, and infrastructure fulfilment to facilitate the implementation practice of a balanced scorecard.

## Background

In the modern world, everything changes over time. As a result, organizations require continual reform initiatives; Kaplan and Norton developed and introduced the concept of BSC in 1992 (1). This BSC concept tries to translate an organization’s strategic direction and objectives into actionable initiatives and measurements (2, 3). In Ethiopia, BSC was started since 2010 in the entire civil service office throughout the country (4).

A performance evaluation study conducted in the UK showed that 70% of BSC implementations fail in all organizations (5). Only around 20% of organizations with a mature BSC implementation are creating business value as a result of their efforts (6), and 5% of the workforce understands the organizations’ strategy (7, 8). Inadequate BSC implementation in health services can lead to poor health service quality and provision, low profit and little improvements (9) and low customer satisfaction as well as retention (9–12).

Studies conducted on BSC practice in Addis Ababa and Australia indicated that age, educational status, work experience (5, 13), vision, mission, performance evaluation, objective, planning, strategic thinking, strategic mapping, infrastructure, working environment, teamwork, and resource availability of job description were found significant factors for BSC implementation practice (1, 14).

The majority of employees in the office are currently unaware of BSC and its practice (13, 15). At the turn of the century, when the value was produced from the most effective use of physical assets and employees were spoken in the great organizational wheel, this condition served (16, 17). Organizations assume that putting BSC into practice can be done quickly and easily after deciding to do so (18).

BSC is a tool that organizations may use to improve information flow and communication with internal and external partners (19, 20). To achieve the efficient application of BSC, all participants in the public sector should be involved in the planning process for the internalization of strategic objectives (21, 22) and should communicate this timely to all health leaders and employees of the organizations (23, 24).

When an organization’s strategic planning is updated at the institutional level, health leaders are expected to realign all of the organization’s personnel with the new direction (25, 26). A critical element was allocating enough resources and successfully applying BSC in hospitals (27) focusing on strategy necessitates (28–30). However, the implementation of BSC is poor and a study in Ethiopia focusing on the application of BSC and its related aspects has been limited. Hence, this study aimed to address this gap by examining the implementation of the BSC and its factors among healthcare workers working in public primary hospitals of the Central Gondar zone.

## Methods

### Study design and setting

A mixed methods study was carried out from May 25 to June 26, 2022. The study took place within the Central Gondar Administrative Zone, located in the Amhara National Regional State, Northwest Ethiopia. Gondar City serves as the administrative seat of this zone, located approximately 750 kilometers away from Ethiopia’s capital, Addis Ababa. In 2021, the projected population of the Central Gondar Zone was 2,307,773, with 1,246,197 being male. Central Gondar zone has nine public primary hospitals with 843 health professionals serving in those public primary hospitals (31).

### Eligibility of the study

The source population were all healthcare professionals who were working at public primary hospitals in the Central Gondar zone. All healthcare professionals in those institutions who had more than six months of work experience were included while those who were contract employees and/or voluntarily serving health professionals were excluded from the study.

### Sample size and sampling procedure

The adequate sample size was determined using the single population proportion formula considering a 95% confidence interval (CI) of Z *α*/2 = 1.96; a margin of error of 0.05; a non-response rate of 10%; and, a 50% magnitude of BSC implementation practice (no previous study). As a result, the sample size used for this study was calculated as follows:

$n=\frac{{\left(Za/2\right)}^{2}p\left(1-p\right)}{d^{2}}$ = 385

By adding a 10% non-response rate the final sample size was 424 study subjects.

There are nine primary hospitals in the central Gondar zone. The sample size for each of these hospitals was determined based on the number of healthcare professionals they have. This ensured proportional representation. To select those study participants, we used a systematic random sampling technique. In addition, in the qualitative part, 12 participants were purposively selected from public primary hospitals based on their experience, knowledge, and year of service related to the interest of the study.

### Study variables

Implementation practice of BSC was the dependent variable, and Socio-demographic variables: (sex, age, education, work experience, profession, marital status, religion, and monthly income), organizational factors: (BSC plan, work environment, an opportunity for training, vision, mission, objective, organization communication, availability of a job description, availability of guidelines, teamwork, resources, infrastructure) and Individual factors: (leader’s and health professional attitude towards BSC, understanding of BSC) were the independent variables.

### Operational definition

Balanced Scorecard is a strategic management performance standard of measurement that helps primary hospitals identify and improve their healthcare needs to become productive and healthy citizens (32).

Good implementation of BSC practice: The measurement was done by using a ten-item outcome measuring tool. If the overall score is above the mean, we said that there is good implementation practice of BSC (by doing eight items on the Likert scale) (11).

### Data collection procedure

A pre-tested structured questionnaire was used to collect the quantitative part and the participants completed the questionnaire independently. For the qualitative part, face-to-face interviews using a pre-tested semi-structured questionnaire were used and the principal investigator undertook the data collection by arranging a comfortable time and place, conducted in Amharic. The information is recorded, transcribed then translated. Afterwards, the responses were transcribed into Amharic, translated into English, and entered into Open Code version 4.03 for thematic analysis. Quantitative and qualitative data were gathered simultaneously, analyzed independently, and merged during the results phase.

### Data quality assurance

Eight Bachelor of Science in Nursing (BSc Nursing) professionals were recruited to assist in administering the questionnaire and the investigator conducted key informant interviews. The questionnaire underwent a preliminary trial with 44 (10%) healthcare workers from a comparable health facility in the Debark Hospital, located outside the study area. Adjustments were made based on the feedback received. A reliability assessment, measured through Cronbach’s Alpha, indicated a score of 0.81 for the internal consistency of the individual items and 0.93 for the dependent items. Before starting the data collection, the data collectors undertook a comprehensive two-day training session for clarity, understanding the objective and management of the questionnaire. Throughout the data collection process, a supervisor oversaw operations, ensuring data completeness and promptly addressing any issues encountered at the data collection sites.

### Data management

Before entering the data into Epi Data version 4.6 software, the data were cleaned and checked for completeness and consistency and then exported to Stata version 14 for further data analysis. For qualitative analysis, the open code version 4.03 software was used. Preceding the analysis phase, the data underwent procedures such as editing, verification, cleansing, coding, and merging, as required, to render it appropriate for analysis.

Descriptive variables were analyzed using percentages and frequencies, while continuous variables were described using the mean and standard deviation. The qualitative data was transcribed and translated using Open Code version 4.03. Before thematic analysis, data cleaning and labelling were carried out.

### Statistical analysis

Binary logistic regression was done and assumptions for the model fitness were checked. Then, bi-variable analyses were conducted to examine the relationship between each independent variable and the dependent variable (the implementation practice of BSC). Variables with *p*-value ≤0.2 in bi-variable analyses were fitted for multivariable logistic regression.

Variables having a *p*-value of less than 0.05 in the multivariable model were considered to have statistical significance associated with the outcome variable. For the qualitative part, we transcribed and translated and thematic analysis was employed by using open code version 4.03 software.

## Results

### Socio-demographic characteristics of study participants

Overall, 404 health professionals participated in the study. The sample consisted of 280 males and 124 females. Among our study participants, 327(80.9%) were in the age group of 26–35 years. The mean age of study participants was 30 (SD 3.8) years. Three hundred thirty-three (97.3%) of the study participants were Orthodox Christians with 251 (62.1%) of the study subjects having first-degree professions. One hundred twenty-six (32.1%) of the study participants were nurses, and 231 (57.2%) of them had ≤5 years of work experience (Table 1).

**Table 1:** Respondent sociodemographic characteristics of the BSC implementation practice in public primary hospitals, Central Gondar zone, North West Ethiopia, 2022 (*N* = 404).

Characteristics	Category	Frequency (%)
Sex	Female	124 (30.7)
	Male	280 (69.3)
Age	18–25	37 (9.2)
	26–35	327 (80.9)
	36–45	40 (9.9)
Service in year	1–5 year	231 (57.2)
	6–10 year	141 (34.9)
	≥11 year	32 (7.9)
Educational status	Diploma	136 (33.7)
	Degree	251 (62.1)
	MSc and above	17 (4.2)
Profession	Medical doctor	24 (5.9)
	Nurse	126 (31.2)
	Laboratory	68 (16.8)
	Pharmacist	39 (9.7)
	Midwifery	49 (12.1)
	Other	98 (24.3)
Religion	Orthodox	393 (97.3)
	Muslim	11 (2.7)
Marital status	Married	278 (68.8)
	Unmarried	123 (30.4)
	Divorced	3 (0.7)
Monthly income	≤4,609	91 (22.5)
	4,610–7,071	223 (55.2)
	≥7,072	90 (22.3)

Others * other than the listed professions such as radiologist, physiotherapist, health informatics.

### Factors affecting the implementation practice of BSC

#### Individual level factors

More than half of the respondents, 232 (57.4%), responded that they did not have a clear understanding of BSC implementation practice, and 254 (55.4%) participants responded that they had a negative attitude towards BSC implementation practice (Table 2).

**Table 2:** Response on the individual factor to the BSC implementation practice in public primary hospital, Central Gondar zone, Northwest Ethiopia May, 2022 (*N* = 404).

Variables	Category	Frequency (%)
Individual understands BSC	Yes	172 (42.6)
	No	232 (57.4)
Individual attitude to BSC implementation practice	Negative	224 (55.4)
	Positive	180 (44.6)

#### The organizational level factors

Regarding organizational communication, 225 (55.7%) of the respondents responded that there was poor organizational communication in their public primary hospitals. Another 226 (55.9%) of the participants responded that teamwork was implemented in their respective public primary hospitals. In terms of infrastructure, 227 (56.2%) of participants expressed that infrastructure was a challenge for the implementation practices of BSC (Table 3).

**Table 3:** Response on the organizational factors in the BSC implementation in public primary hospital, Central Gondar zone, Northwest Ethiopia May 2022 (*N* = 404).

Variables	Category	Frequency (%)
Opportunity for individual growth and development	No	245 (60.6)
	Yes	159 (39.4)
Teamwork in BSC implementation	Implemented	226 (55.9)
	Not implemented	178 (44.1)
Clarity of the of the mission	Not clear	272 (67.3)
	Clear	132 (32.7)
Clarity of the vision	Not clear	202 (50)
	Clear	202 (50)
Work environment	Not comfortable	226 (55.9)
	Comfortable	178 (44.1)
Organization communication	Poor	225 (55.7)
	Good	179 (44.3)
BSC implementation plan strategy	Not clear	214 (53)
	Clear	190 (47)
Job description of the primary hospital	Not available	125 (30.9)
	Available	279 (69.1)
Objective of primary hospital	Not clear	298 (73.8)
	Clear	106 (26.2)
Resource for BSC implementation	Not allocated	248 (61.4)
	Allocated	156 (38.6)
Availability of BSC implementation guide line	Not available	265 (65.6)
	Available	139 (34.4)
BSC performance evaluation period	No appropriate	234 (57.9)
	Appropriate	170 (42.1)
Infrastructure	Not available	227 (56.2)
	Available	177 (43.8)

### Magnitude implementation practice of BSC in public primary hospitals

The percentage of overall implementation practice of BSC in the central Gondar zone was found at 48.5% (95% CI, 43.6, 53.4) (see Table 4).

**Table 4:** Binary and multi variable logistic regression on the factors associated with balanced scorecard implementation practice in Central Gondar zone Ethiopia 2022.

Variables	Category	Poor BSC practice	Good BSC practice	COR (95%CI)	AOR	
					(95%CI)	*p*-value
Sex	Female	50	74	1	1	
	Male	158	122	0.52 (0.34,0.80)	0.65 (0.36,1.17)	0.15
Individual attitude to implementation BSC	Negative	144	80	1	1	
	Positive	64	116	13.26 (2.17,4.91)	1.29 (0.71,2.33)	0.4
Opportunity for training	No	152	93	1	1	
	Yes	56	103	3.01 (1.98,4.55)	0.88 (0.48,1.60)	0.67
Teamwork implemented	No	151	75	1	1	
	Yes	57	121	4.27 (2.81,6.5)	2.68 (1.53,4.69)	0.01*
Mission of organization	Not understand	160	112	1	1	
	Understand	48	84	2.5 (1.63,3.84)	0.66 (0.33,1.31)	0.23
Vision of organization	Not clear	126	76	1	1	
	Clear	82	120	2.43 (1.63,362)	0.54 (0.26,1.11)	0.09
Work environment	Not comfortable	156	70	1	1	
	Comfortable	52	126	5.4 (3.52,8.29)	1.31 (0.69,2.48)	0.41
Communication	Poor	159	66	1	1	
	Good	49	130	6.39 (4.13,9.89)	3.24 (1.79,5.89)	0.01*
BSC plan stratagem	not based on plan	152	62	1	1	
	Based on plan	56	134	5.87 (3.82,9.01)	1.84 (0.93,3.64)	0.08
Job discretion in the primary hospital	Not available	88	37	1	1	
	Available	120	156	3.15 (2.01,4.95)	1.43 (0.77,2.64)	0.26
Objective primary hospital	disagree	167	131	1	1	
	agree	41	56	2.02 (1.28–3.18)	0.85 (0.42,1.75)	0.67
Resource	Not available	160	88	1	1	
	Available	48	108	4.09 (2.67,6.28)	1.36 (0.72,2.57)	0.34
BSC implementation guideline	Not available	170	95	1	1	
	Available	38	101	4.76 (3.03,7.46)	1.97 (0.99,3.92)	0.06
BSC evaluation period	Not appropriate	158	76	1	1	
	Appropriate	50	120	4.99 (3.25,7.66)	2.48 (1.32,4.67)	0.08
Infrastructure available	Not available	145	82	1	1	
	Available	63	196	3.2 (2.12,4.82)	2.03 (1.14,3.64)	0.02*

COR: Crude odds ratio, CI: Confidence interval, AOR: adjusted odds ratio, 1: Reference category, * *p*-value < 0.05.

### Factor associated with the BSC implementation practice

Candidate variables identified from bi-variable regression for multivariable logistic regression were sex, individual attitude, the opportunity for training, teamwork, the mission of the organization, vision of the organization, work environment, organizational communication, agreement on BSC plan and strategy, job description, objective of hospitals, resource availability and, infrastructure availability.

The odds of good organizational communication were 3.24 times (AOR: 3.24 at 95% CI: 1.79, 5.89) higher odds of implementation practice of BSC compared to poor organizational communication. This finding is also supported by qualitative findings. Most of the key informants agreed that there was poor organizational communication that caused poor implementation practices of BSC.

"*In our hospital, there is suboptimal organizational communication among health professionals and with other stakeholders, which leads to the poor implementation practice of BSC. Effective communication is essential, as it positively influences health professionals' attitudes and behaviors, ultimately contributing to better implementation practice of BSC." (A 33-year-old case team coordinator at KI4)*

"*In this organization, there is a problem with effective communication and information-sharing culture between leaders and professionals. These concerns hamper the implementation of BSC and negatively influence the overall performance of the hospital.” (A 31-year-old hospital case manager, KI 6)*

The odds of implementing teamwork 2.68 (AOR; 95% CI; 1.53, 4.69) were higher odds of implementation practice of BSC over those who did not work in teamwork. This result was supported by our qualitative findings. Information obtained via key informant interviews indicates that implementing teamwork enhances the implementation of BSC. Most of the key informants agreed that teamwork is a facilitator for implementing BSC in their hospitals.

“*Most of the tasks in our hospital are done in group performance. This includes activities like morning sessions and general meetings of the hospital. Clients are managed by teamwork, involving many individuals throughout the process, from diagnosis to treatment. Therefore, teamwork is essential in a healthcare setting to align team goals with organizational strategy, establish clear performance metrics, and ensure continuous improvement. However, I don't believe that teamwork is effectively implemented in our hospital. This may be a significant factor contributing to the poor implementation of the BSC" (A 32-year-old medical director at KI1)*.

The odds of the availability of infrastructures were 2.03 (AOR: 95%, CI 1.14, 3.64) higher odds of implementation practice of BSC compared to the absence of infrastructure. The result was in line with a qualitative finding. Because most key informants agreed, that there is a lack of infrastructure facilities for effectively implementing BSC practice. *A 31-year-old hospital manager at KI 3 said:*

"*There are various infrastructure obstacles hindering the effective performance of BSC. These obstacles are insufficient internet service; lack of a complete electric system to deliver quality service, and bring customer satisfaction; and no computers for updated health information recording and use by workers to implement BSC. As a result of this, "our BSC implementation is not as much as it is expected to be performed*."

Most of the key informants agreed and reported as:

“*The challenges identified in the effective implementation of BSC were: lack of commitment from management and health professionals; a negative attitude towards BSC; scarcity of human and financial resources; lack of an updated job description; inappropriate evaluation periods; inconsistency in support and monitoring mechanisms; poor organizational communication; lack of infrastructure and absence of BSC training. Lack of accountability and lack of recognition are some of the most common issues faced by health professionals to implement BSC*”

"*We can explain that among so many barriers in the BSC implementation, some of them are lack of motivation, lack of training evaluation, feedback, low support, poor revenue collection, poor budget utilization, and lack of ability to generate new ideas from the health professionals" (A 32-year-old hospital metherone KI4)*

"*When we come back to the hurdle of BSC implementation practice, there are barriers such as attendance to keep as traditional, knowledge gap, lack of commitment, lack of attention, and follow-up," (A 32-year-old hospital manager KI2)*

## Discussion

BSC implementation practice is valuable particularly for primary hospital settings, because of different reasons. It helps the organization to manage resources effectively and efficiently, improve patient care, and achieve strategic goals. This study has revealed that the magnitude of the implementation practice of BSC was 48.5%. The finding is very low compared to the national standards (33) and previous study findings in Ethiopia (34). The reason for this low implementation might be that the qualitative study conducted in Scandinavian on BSC users showed four main problem areas associated with the implementation of the BSC concept, such as conceptual, technical, social, and political issues that affect the implementation of BSC (35, 36).

On the other hand, a study conducted in England on the use of BSC in health care settings showed that 70% of BSC implementations failed in all health sectors. The possible reasons identified in this study for this poor implementation have been attributed to two main causes: poor design and implementation issues (37). With this research, the focus was on the implementation aspect of BSC, and several problems were witnessed, the cause of poor implementation practice in the study area was observed from the implementation practice of BSC. In addition to our qualitative results, the data obtained from the interview stated that a lack of commitment from management and health professionals; inconsistency in support and monitoring; and limited resources for BSC implementation led to poor implementation practices of BSC. This might be the reason for the low implementation of BSC in this study area.

According to the study findings, working in a team was 2.68 times more likely to improve the implementation practice of BSC as compared to those working individually. The study conducted in Iran supports this finding. It shows the direct significant effect of teamwork on BSC implementation practice (37). Encouragement of teamwork has an important contribution to the improvement of health organizations’ BSC performance and also helps to overcome individual limitations synergistically. Problems can be solved through a teamwork spirit (37). To reinforce these concepts, the literature suggests that a key challenge for managers is to motivate their teams to collaborate effectively as a cohesive unit, rather than functioning as individuals focused solely on their tasks (38).

Good organizational communication for BSC implementation was found 3.24 times more likely to have good implementation practice than those who had poor organizational communication. The importance of good organizational communication toward BSC implementation is indicated in different kinds of literature results. To ensure effective implementation of BSC, all the participants in public primary hospitals should be involved in the planning process for internalization of the strategic objectives and should communicate timely to address the information gaps to all leaders and employees of the organizations. BSC is used to improve the flow of information within organizations and to advance communication with internal and external partners. One of the reasons for poor implementation practice of BSC is that organizations do not communicate BSC throughout the entire organization, undermining its essence (39). On the other hand, a recent survey conducted in jurymen on the existing challenges of BSC implementation found insufficient information flow systems to support BSC implementation (14, 40). On top of these, there is also another concept that confirms our findings. Organizational communication is the highest priority and the first strategy required for any organizational change management through BSC implementation. It reduces the restrain by keeping health professionals informed about what to expect from the change effort.

In this study, the availability of infrastructure facilities in public primary hospitals was 2.03 times more likely to practice BSC implementation than those hospitals with a lack of infrastructure facilities. This finding is supported by the study conducted by Othman: weak infrastructure facility, such as lack of good office layout, is supported by a weak IT infrastructure and automated data collection (23). On the other hand, one of the common challenges of implementing BSC is infrastructure, such as inadequate IT support (41). A smooth implementation of any BSC system should be supported by appropriate infrastructure accessibility. Health organizations’ pre-determined BSC goals and objectives need to be supported by well-furnished infrastructure facilities on a timely basis (42). Furthermore, the availability of infrastructure inputs enhances the learning process, and also improves the internal business processes of the organization; that improvement leads to improved customer satisfaction and quality health service provision (43).

## Strengths and limitations of the study

This study applied quantitative approaches supported by a qualitative finding. This mixed study provided an excellent opportunity to explore the challenges of BSC implementation practice. The research carried out in this thesis is cross-sectional and has faced several limitations, such as causal relationships between factors of the BSC and the implementation practice of the BSC, and the data collection method was self-reported, it was subjected to response bias. This program is implemented throughout the nation, therefore in the future, the researcher will carry out as a nation and out of primary hospitals.

## Conclusion

The findings of this study indicated that the extent of BSC implementation was inadequate. Through statistical verification, we confirmed that key factors; such as organizational communication, teamwork, and infrastructure; are significantly related to the implementation practices of the BSC in the public primary hospitals surveyed. The results of this research provide a foundation for further analysis and identification of factors influencing the implementation of BSC in the health sector.

As our qualitative study findings indicated, the challenges for successful BSC implementation were: lack of commitment from management and health professionals; negative attitude towards BSC; scarcity of human and financial resources; lack of updated job description; an inappropriate evaluation period; inconsistency in support and monitoring; poor organizational communication; lack of infrastructure facility; and a lack of BSC training opportunity.

## Data Availability

The raw data supporting the conclusions of this article will be made available by the authors, without undue reservation.
